# The Pyrazolo[3,4-d]pyrimidine Derivative, SCO-201, Reverses Multidrug Resistance Mediated by ABCG2/BCRP

**DOI:** 10.3390/cells9030613

**Published:** 2020-03-04

**Authors:** Sophie E. B. Ambjørner, Michael Wiese, Sebastian Christoph Köhler, Joen Svindt, Xamuel Loft Lund, Michael Gajhede, Lasse Saaby, Birger Brodin, Steffen Rump, Henning Weigt, Nils Brünner, Jan Stenvang

**Affiliations:** 1 Department of Drug Design and Pharmacology, University of Copenhagen, 1353 Copenhagen, Denmark; sophie.ambjoerner@gmail.com (S.E.B.A.); lxs184@alumni.ku.dk (X.L.L.); mig@sund.ku.dk (M.G.); nb@scandiononcology.com (N.B.); 2 Pharmaceutical Institute, University of Bonn, 53012 Bonn, Germany; mwiese@uni-bonn.de (M.W.); skoehler@uni-bonn.de (S.C.K.); 3 Department of Biology, University of Copenhagen, 1353 Copenhagen, Denmark; joen.svindt@gmail.com; 4 Department of Pharmacy, University of Copenhagen, 1353 Copenhagen, Denmark; lasse.saaby@sund.ku.dk (L.S.); birger.brodin@sund.ku.dk (B.B.); 5 SRConsulting, 31319 Sehnde, Germany; rump@s-r-consulting.com; 6 Division of Chemical Safety and Toxicity, Fraunhofer Institute of Toxicology and Experimental Medicine, 30625 Hannover, Germany; henning.weigt@item.fraunhofer.de; 7 Scandion Oncology A/S, Symbion, 1353 Copenhagen, Denmark

**Keywords:** multidrug resistance in cancer, drug efflux pumps, ATP-binding cassette transporter, breast cancer resistance protein (BCRP), ABCG2, pyrazolo-pyrimidine derivative, SCO-201

## Abstract

ATP-binding cassette (ABC) transporters, such as breast cancer resistance protein (BCRP), are key players in resistance to multiple anti-cancer drugs, leading to cancer treatment failure and cancer-related death. Currently, there are no clinically approved drugs for reversal of cancer drug resistance caused by ABC transporters. This study investigated if a novel drug candidate, SCO-201, could inhibit BCRP and reverse BCRP-mediated drug resistance. We applied in vitro cell viability assays in SN-38 (7-Ethyl-10-hydroxycamptothecin)-resistant colon cancer cells and in non-cancer cells with ectopic expression of BCRP. SCO-201 reversed resistance to SN-38 (active metabolite of irinotecan) in both model systems. Dye efflux assays, bidirectional transport assays, and ATPase assays demonstrated that SCO-201 inhibits BCRP. In silico interaction analyses supported the ATPase assay data and suggest that SCO-201 competes with SN-38 for the BCRP drug-binding site. To analyze for inhibition of other transporters or cytochrome P450 (CYP) enzymes, we performed enzyme and transporter assays by in vitro drug metabolism and pharmacokinetics studies, which demonstrated that SCO-201 selectively inhibited BCRP and neither inhibited nor induced CYPs. We conclude that SCO-201 is a specific, potent, and potentially non-toxic drug candidate for the reversal of BCRP-mediated resistance in cancer cells.

## 1. Introduction

**Figure 1:**
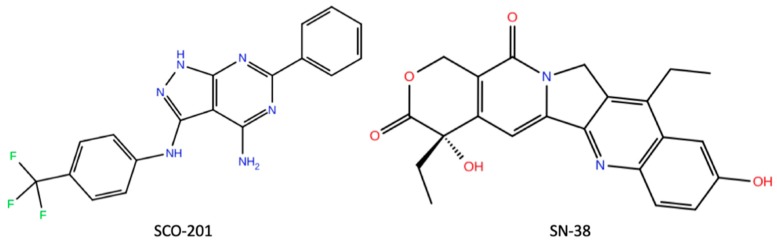
Chemical structures of the pyrazolo[3,4-d]pyrimidine derivative SCO-201 and the active metabolite of irinotecan, SN-38. Graphics produced using Maestro, Schrödinger 2019-3, limited liability company (LLC), New York, NY, 2019. SN-38 structure obtained from PubChem Database [35,36].

Chemotherapy resistance is considered the single most important obstacle to greater success with chemotherapy for cancer patients [1,2,3]. Although many cancer patients initially benefit from chemotherapy treatment, a large proportion of treatments fail due to acquisition of resistance to multiple anti-cancer drugs. This phenomenon is known as multidrug resistance (MDR) and refers to the concurrent development of cross-resistance to many chemically diverse anti-cancer agents [4]. MDR results in poor prognosis and decreased survival rate of cancer patients, and strategies to circumvent MDR are therefore highly needed [3,5]. The mechanisms underlying MDR are complex and include many different tumor survival mechanisms [6]. Overexpression of drug expelling ATP-binding cassette (ABC) transporters seems to be an important mechanism of MDR in cancer cells [7]. Today, the most extensively studied and characterized ABC transporters, found to be involved in cancer MDR, are (a) multidrug resistance protein 1/P-glycoprotein (MDR1/P-gp), encoded by the *ABCB1* gene; (b) multidrug resistance-associated protein 1 (MRP1), encoded by the *ABCC1* gene; and (c) breast cancer resistance protein (BCRP), encoded by the *ABCG2* gene [8]. ABC transporters are normally expressed in tissues such as the intestines, brain, liver, and placenta, where they prevent xenobiotic substrates from accumulating [7]. The ABC transporters are transmembrane proteins that utilize ATP hydrolysis to drive the active transport of substrates from the cytoplasmic site to the extracellular space [9]. The transporters consist of two transmembrane domains (TMDs), able to undergo a conformational change that triggers the removal of the substrate, and two cytoplasmic nucleotide-binding domains (NBDs) that bind and hydrolyze ATP [10]. Due to a broad drug specificity, ABC transporters can efflux many different anticancer agents, thus resulting in MDR [7,9]. BCRP (ABCG2) is a 72 kDa half-transporter that acts as a homomeric dimer, and so far, BCRP is known to mediate resistance to a variety of anti-cancer agents, among these the chemotherapeutic agents SN-38, topotecan, mitoxantrone, doxorubicin, and daunorubicin [11,12,13,14,15,16]. SN-38 (Figure 1) is the active metabolite of irinotecan (Camptosar) and is especially important in the treatment of gastrointestinal cancers such as colorectal cancer [17] and pancreatic cancer (European Society for Medical Oncology (ESMO) guidelines for pancreatic cancer). Several studies have indicated that high cancer cell levels of BCRP is the key player in SN-38 resistance, and BCRP thus hinders successful treatment of metastatic gastrointestinal cancer patients [11,12,13,14,15,16]. Mitoxantrone was the first chemotherapy to be identified as a substrate of BCRP, and BCRP was found to be involved in mitoxantrone-resistant breast cancer, thus giving BCRP its name [13]. 

During the last 40 years, researchers have tried to develop non-toxic, highly potent, and efficacious drugs that are able to reverse ABC-transporter-mediated MDR [7,9,17,18,19]. These MDR-reversing agents, also known as re-sensitizing agents or chemo-sensitizers, act by either inhibiting the expression of ABC transporters or by directly inhibiting the transport function, and thereby restore the sensitivity of the cancer cells to anti-cancer agents [9,10]. The compound fumitremorgin C was the first BCRP inhibitor to be identified, and although it was found to have a high inhibitory potency, neurotoxic side effects prevented the clinical use of this compound [20,21]. To prevent these side effects, researchers synthesized new different fumitremorgin C analogues, for instance, the potent BCRP inhibitor Ko143 [22,23]. Nonetheless, these analogues, including Ko143, were not stable in plasma, still caused the side effects, and could not be used in the clinic [23]. Other known ABC transporter inhibitors include verapamil, tariquidar, and valspodar (PSC833), which all inhibit MDR1/P-gp [9]. However, despite a long list of different potent inhibitors, none of these have been approved for clinical use. The lack of ABC transporter inhibitors in clinical use can be attributed to several issues: (1) the inhibitors specifically only inhibit one transporter, (2) the inhibitors exhibit a significant degree of toxicity, (3) clinical studies were poorly designed—inhibitors were not combined with the drug that the patients had proved to be resistant to—and the studies lacked randomization, and (4) lack of companion diagnostic tests to optimize patients’ selection and treatment [1,7,9]. Thus, new strategies are greatly needed to improve the treatment success and survival rate of cancer patients with MDR.

To identify potential new compounds that interfere with common drug resistance mechanisms, such as the overexpression of BCRP, we previously established the DEN-50R screening platform. This platform consists of isogenic pairs of drug-sensitive and drug-resistant patient-derived cancer cell lines, for instance, colorectal, breast, prostate, and pancreatic cancer [24]. These resistant cell lines were established by exposing chemotherapy-sensitive cells to gradually increasing concentrations of chemotherapy over a period of 8–10 months [25]. We thoroughly characterized these drug-resistant cell lines to identify important drug resistance mechanisms [25,26,27,28,29,30]. In accordance with several other studies with in vitro model systems [31], we found that BCRP overexpression was a key player of resistance to SN-38 [25]. Using our DEN-50R screening platform, we found that pyrazolo-pyrimidine derivatives might serve as potential inhibitors of drug resistance in these cell lines. One of the hits from the drug screening was the pyrazolo-pyrimidine derivative SCO-201 (previously OBR-5-340) (Figure 1). These preliminary data indicated that SCO-201, which is previously known as a potent viral capsid inhibitor, might serve as a potential inhibitor of drug resistance [32,33]. Interestingly, in a study by Burkhart et al. (2009) [34], it was demonstrated that pyrazolo-pyrimidine derivatives comprise a prominent structural class of selective and potent ABC transporter inhibitors, with low toxicity and low risk of increased chemotherapy-mediated toxicity [19,34].

On the basis of these findings, the aim of this study was to clarify if SCO-201 re-sensitizes cancer cells to chemotherapy substrates of BCRP. An additional aim was to investigate any potential risks of pharmacokinetic interactions of SCO-201, which was investigated by testing the effect of SCO-201 on cytochrome P450 enzymes and efflux/uptake transporters. Our study is the first to show that SCO-201 competitively inhibits the transport activity of BCRP, triggers the accumulation of BCRP dye substrate, and re-sensitizes cancer cells to chemotherapy in two different in vitro models of BCRP-mediated resistance. Moreover, in vitro drug metabolism and pharmacokinetics (DMPK) data suggest that SCO-201 will not influence metabolism of other drugs by the cytochrome P450 (CYP) system. This indicates that SCO-201 is not likely to increase the risk of pharmacokinetic interactions with chemotherapy that are metabolized by the CYP system. The present data warrant further studies including regulatory toxicity and ADME (absorption, distribution, metabolism, and excretion) studies according to good laboratory practice (GLP) in order to initiate clinical development [35,36].

## 2. Materials and Methods

### 2.1. Reagents and Antibodies

DMSO, SN-38, Ko143, topotecan, mitoxantrone, Hoechst 33342, MTT reagent (3-(4,5-dimethylthiazol-2-yl)-2,5-diphenyltetrazolium bromide), primary antibody for β-actin (mAb A5441), PREDEASY SB-MDR1/P-gp Hi5, and SB-BCRP-M ATPase assays kits (Solvo Biotechnology) were all purchased from Sigma-Aldrich/Merck (Schnelldorf, Germany). PSC833 was purchased from Tocris/Bio-techne (Abingdon, UK). The primary antibody for BCRP (mAb BXP-21) was purchased from Abcam (Cambridge, UK). 

Plasticware, such as T-75 culture flasks and Transwell permeable supports (1.12 cm^2^, 0.4 µm pores), were purchased from Corning, Fisher Scientific (Slangerup, Denmark). Cell culture reagents such as Hank’s balanced salt solution (HBSS) was purchased from Life Technologies (Taastrup, Denmark); fetal bovine serum (FBS) from Gibco, Fischer Scientific (Slangerup, Denmark); penicillin and streptomycin were acquired from Bio Whittaker Cambrex (Vallensbaek, Denmark); Dulbecco’s modified Eagle’s medium, bovine serum albumin (BSA), and Minimum Essential Media (MEM) nonessential amino acids were purchased from Sigma-Aldrich (Brøndby, Denmark); whereas 2-[4-(2-hydroxyethyl)piperazin-1-yl] ethanesulfonic acid (HEPES) was purchased from AppliChem GmbH (Darmstadt, Germany). [3H]-Estrone-3-sulfate (51.8 Ci/mmol), [14C]-mannitol (0.06 Ci/mmol), and Ultima Gold scintillation fluid was purchased from PerkinElmer (Boston, MA, USA).

All drugs were dissolved in DMSO, except mitoxantrone, which was dissolved in ethanol.

### 2.2. Cell Lines and Culture Conditions

All cell lines were maintained at 37 °C in a humidified 5% CO_2_ atmosphere. The Madin-Darby Canine Kidney (MDCK)-II-BCRP cell line was a kind gift from Dr. A. Schinkel (The Netherlands Cancer Institute, Amsterdam, The Netherlands [29,30]) and cultured in Dulbecco’s modified Eagle’s medium (DMEM) supplemented with 10% fetal calf serum (FCS), 50 µg/mL streptomycin, 50 U/mL penicillin G, and 2 mM l-glutamine. The parental drug-sensitive (HT29_PAR_), obtained from the National Cancer Institute (NCI)/Development Therapeutics Program, and SN-38-resistant (HT29_SN-38-RES_) cell lines were cultured in Gibco Roswell Park Memorial Institute (RPMI) 1640–GlutaMAX medium supplemented with 10% FCS [25].

### 2.3. Western Blot Analysis

The proteins of HT29 cell lysates were separated with SDS-PAGE and transferred to nitrocellulose membranes. After blocking the membrane with 5% skimmed milk in 1× Tris-Buffered Saline, 0.1% Tween^®^ 20 Detergent (TBS-T), the membrane were incubated with primary monoclonal antibodies: anti-BCRP (BXP-21; 1:1000) and β-actin (1:500,000) overnight at 4 °C, and thereafter with HRP-conjugated secondary antibody (1:4000) for 1 h. Bands were detected using Enhanced Chemiluminescence (ECL) peroxide solution and luminol/enhancer solution (Clarity Western ECL Substrate, Bio-Rad Laboratories (Copenhagen, Denmark)) for 5 min. Images were obtained with the UVP Biospectrum imaging system (VisionWorks software, version LS 7.0.1). 

### 2.4. MDR Reversal Analysis with MTT Assay

Cells were seeded in Nunc 96-well plates (2000 or 8000 cells/well for the MDCK-II and HT29 cells, respectively). Following cell attachment (12–24 h), drugs were added to a total volume of 200 µL. Control conditions consisted of full growth medium. All treatments were performed in triplicate. Following 72 h of drug exposure, MTT reagent was added for either 1 h (MDCK-II) or 3 h (HT29). For cell lysis and solubilization of the formazan crystals, DMSO was added to the MDCK-II cells, whereas acidified (0.02 M HCl) sodium dodecyl sulphate was added to the HT29 cells. Optical densities were measured with a microplate spectrophotometer at either 544 nm and 710 nm (background) for the MDCK-II cells or 570 nm and 670 nm (background) for the HT29 cells. Background optical density values were subtracted, and the average optical densities were calculated. Cell viability was calculated as percentage of untreated control cells. The mean IC_50_ values were determined using GraphPad Prism (version 6.0, San Diego, CA, USA). The drug sensitivity analysis was carried out at least three independent times and representative data is shown. 

### 2.5. Bidirectional Transport Assay

Bidirectional transport experiments with [3H]-estrone-3-sulfate were completed with monolayers of Caco-2 cells from the American Type Culture Collection (ATCC) cultured on Transwell permeable supports for 27 days in Dulbecco’s modified Eagle’s medium, supplemented with 10% FBS, 10 μL·mL^−1^ nonessential amino acids (×100), and 100 U·mL^−1^ to 100 μg·mL^−1^ penicillin-streptomycin solution. The transepithelial electrical resistance was measured across Caco-2 cell monolayers with an Endohm 12-cup electrode chamber (World Precision Instruments Inc., Sarasota, FL, USA) connected volt meter (EVOM, World Precision Instruments Inc., Sarasota, FL, USA) to ensure that cell monolayers were electrically tight before initiating transport experiments. Cell monolayers were allowed to equilibrate to room temperature before the resistance was measured. Prior to initiating the transport experiments, the cell monolayers were pre-incubated in transport buffer (HBSS supplemented with 10 mM HEPES, pH 7.4, and 0.05 % BSA) for 30 min. Transport experiments were started by replacing the blank transport buffer in the donor compartment with transport buffer containing [3H]-estrone-3-sulfate (1 µCi/mL) and [14C]-mannitol (0.8 µCi/mL) with or without 10 µM SCO-201. For transport experiments in the apical to basolateral direction, samples of 100 µL were taken from the basoteral compartment (volume 1 mL) at t = 15, 30, 45, 60, 90, and 120 min. From transport experiments in the basolateral to apical direction, samples of 50 µL were taken from the apical compartment (volume 0.5 mL) at the same time points. The withdrawn sample volume from the acceptor compartments were immediately replaced with an equal volume of blank transport buffer. The withdrawn samples were pipetted into scintillation vials and mixed with 2 mL of scintillation fluid. The radioactivity of the samples was determined by means of liquid scintillation (Packard Tri-Carb 2910 TR, PerkinElmer, Waltham, MA, USA). Transport of mannitol was measured to validate the barrier integrity of the Caco-2 cell monolayers. The overall average apparent permeability of mannitol across Caco-2 cell monolayers was 2.1 ± 0.4 × 10^−7^ cm·s^−1^ (*n* = 3, total *N* = 9), which is within the expected range for mannitol permeability across intact monolayers of Caco-2 cells.

Data treatment: The accumulated amount of compound (*Qt*, nmol) appearing in the donor compartment was plotted against time. The steady-state flux of compound was calculated as the slope of the linear part of this plot, thus correcting for lag-time effects. The apparent permeability was subsequently calculated with Equation (1):

(1) $$P=\frac{J}{C\_0}=\frac{Q\_t}{\left(C_{0}*A\_t\right)}$$

where *J* represents the steady state flux (nmol·cm^−2^·min^−1^), *C_0_* represents the initial concentration in the donor compartment, *A_t_* denotes the area of the permeable support (1.12 cm^2^), and *Q_t_* is the accumulated amount of compound (nmol) in the receiver compartment at time *t* (min).

The ratio between apparent permeability in the basolateral to apical direction and the apparent permeability in the opposite direction (efflux ratio = $\frac{PB-A}{PA-B}$) was used as a measure of active efflux transport. 

### 2.6. Cellular Dye Efflux Assay 

#### 2.6.1. HT29 Cells

HT29 cells were seeded either into a 96-well Nunclon plate (Thermo Fisher Scientific, Roskilde, Denmark) at a density of 6000 cells/well (for Celigo Imaging Cytometry) or into a Nunc 6-well plate at a density of 150,000 cells/well (for fluorescence microscopy). After 24 h incubation for cell attachment, the cells were incubated with either drug, DMSO or medium for 1 h, then stained with 5 µg/mL Hoechst 33,342 and incubated for 1 h at 37 °C. Then, the cells were washed with ice-cold PBS to remove excess Hoechst dye. Drugs were added again and the cells were incubated for 1h at 37 °C. The plates were analyzed with either fluorescence microscopy (6-well plates) or imaging cytometry (96-well plates). For the Celigo Imaging Cytometry (Lawrence, MA, USA), the application “Target 1 + 2 (merge)” was used, and the mean fluorescence intensities were measured and data presented as percentage of parental control. 

#### 2.6.2. MDCK-II-BCRP Cells

The inhibitory effect of SCO-201 on BCRP was determined in the Hoechst 33,342 accumulation assay as described earlier [37]. Briefly, cells were pre-incubated with SCO-201 for 30 min and then Hoechst 33,342 was added to a final concentration of 1 mM. Fluorescence was measured immediately in constant intervals (60 s) for a period of 120 min with an excitation of 355 nm and an emission wavelength of 460 nm at 37 °C using microplate readers (POLARstar and FLUOstar optima by BMG Labtech, Offenburg, Germany). Background fluorescence was subtracted and the average fluorescence between 100 and 109 min obtained in the steady state was calculated and plotted against the logarithm of the compound concentration. Dose–response curves were fitted by nonlinear regression using the four-parameter or three-parameter logistic equation, whichever was statistically preferred (GraphPad Prism, version 6.0, San Diego, CA, USA).

### 2.7. ATPase Assay

The effect of SCO-201 on the ATPase activity of human BCRP was measured using the PREDEASY ATPase assay system (Solvo Biotechnology, (Sigma-Aldrich/Merck, Schnelldorf, Germany). The assay is a modification of the method of Müller and Sarkardi et al. [38], and the procedure was carried out according to the instructions provided by the manufacturer. Briefly, recombinant BCRP membranes (provided by Solvo Biotechnology) were incubated in the presence or absence of vanadate and different concentrations of either SCO-201 or SN-38, and incubated at 37 °C for 10 min. To test the effect of SCO-201 on the sulfasalazine-stimulated ATPase activity of BCRP, the membranes were prepared with sulfasalazine, prior to the incubation with SCO-201. To test the ability of SCO-201 to hinder the stimulation of BCRP by SN-38, the membranes were prepared with either 0.5 or 1.5 µM SCO-201 and then incubated with different concentrations of SN-38. After incubation with test compounds, MgATP was added to each well and incubated at 37 °C for 10 min. The ATPase reaction was stopped by the addition of 1x Developer solution at room temperature (RT). Two minutes after, 100 µL Blocker solution was added and the plate was incubated for 30 min at 37 °C for 30 min. The optical densities were measured at 620 nm using a PowerWave X Microplate spectrophotometer (BioTek, Bad Friedrichshall, Germany). 

### 2.8. Molecular Interaction Modelling

Docking of SCO-201 and SN-38 in human BCRP transporter were performed using Glide, Schrödinger Release, 2019-3, limited liability company (LLC) [39,40,41]. The 3D structure of BCRP was obtained from the Research Collaboratory for Structural Bioinformatics (RCSB) Protein Data Bank (PDB ID: 6ETI) [16] and prepared using Protein Preparation Wizard, Schrödinger 2019-3, LLC [42]. SCO-201 and SN-38 were prepared using Ligprep, Schrödinger 2019-3, LLC, and docked using flexible XP docking with sampling of both nitrogen inversions and ring conformations. Further characterization of the binding between BCRP and the ligands were performed using the Desmond Molecular Dynamics System, D.E. Shaw Research, Schrödinger, 2019-2, LLC [43]. A standard membrane was fitted to the transmembrane domain of the transporter, and the system was saturated with ions and water molecules. Simulation was run for 10ns and analyzed visually and using the Simulations Interactions Diagram, Desmond, Schrödinger, 2019-2, LLC [43]. 2D docking graphics were produced from Desmond, Schrödinger, 2019-2, LLC, and 3D molecular graphic images of docking were established using the PyMOL Molecular Graphics System, Version 2.0, Schrödinger, LLC.

### 2.9. In Vitro DMPK Analysis: Transporter Inhibition Analysis

Cells were seeded in a 96-well plate (20,000 cells/well) and used on days 2 or 3 post-seeding. SCO-201 was prepared in assay buffer (HBSS-HEPES, pH 7.4), added to the cell plate and pre-incubated at 37 °C for 15 min. SCO-201 was tested at either a single concentration (10 µM by default) or multiple concentrations (0.03, 0.1, 0.3, 1, 3, 10, 30, and 100 µM by default), with a final DMSO concentration of 1%. Subsequently, substrate was added to the plate followed by 20 min incubation at 37 °C. The plate was then washed with cold assay buffer followed by fluorescence reading on a plate reader. The tested transporters, cell lines, substrates, and reference inhibitors are shown in the Supplementary Materials (Tables S1 and S2). 

### 2.10. In Vitro DMPK Analysis: CYP Inhibition 

The following procedure was used to asses if SCO-201 inhibits the activity of common CYP enzymes in pooled human liver microsomes in 96-well plate format. SCO-201 was pre-incubated with substrate and human liver microsomes (mixed gender, pool of 50 donors, 0.1 mg/mL) in phosphate buffer (pH 7.4) for 5 min in a 37 °C shaking waterbath. SCO-201 was tested at either a single concentration (10 µM by default) with 0.1% DMSO or multiple concentrations (0.03, 0.1, 0.3, 1, 3, 10, 30, and 100 µM by default) with up to 1% DMSO for IC_50_ determination. The reaction was initiated by adding a Nicotinamide adenine dinucleotide phosphate (NADPH)-generating system. The reaction was allowed for 10 min and stopped by transferring the reaction mixture to acetonitrile/methanol. Samples were mixed and centrifuged. Supernatants were used for HPLC-MS/MS of the respective metabolite. Tested CYP enzymes, substrates, metabolites, and reference inhibitors are shown in the Supplementary Materials (Tables S2 and S3).

Data analysis: Peak areas corresponding to the metabolite were recorded. The percent of control activity was calculated by comparing the peak area in the presence of the test compound to the control samples containing the same solvent. Subsequently, the percent inhibition was calculated by subtracting the percent control activity from 100. The IC_50_ value (concentration causing a half-maximal inhibition of the control value) was determined by non-linear regression analysis of the concentration–response curve using the Hill equation.

### 2.11. In Vitro DMPK Analysis: CYP Induction

The following procedure was carried out to test whether SCO-201 induces CYP1A, CYP2B6, and CYP3A activities in human hepatocytes. The procedure was designed in accordance with the FDA Guidance for Industry on Drug Interaction Studies (2006). Male and female human hepatocytes were thawed and plated into collagen-coated 96-well plates in serum-containing medium (plating medium) at a density of 0.7 × 10^6^ viable cells/mL. The hepatocytes were cultured in a humidified incubator at 37 °C and 5% CO_2_. At 4 h post plating, human hepatocytes were washed once with fresh plating medium, followed by overnight incubation. At 24 h after plating, the plating medium was removed, and the hepatocytes were overlaid with extracellular matrix (ECM) (Sigma) or Matrigel (BD) in the serum-free medium (incubation medium), and then incubated for another 24 h. Incubation medium with 0.1% DMSO was used as the negative control. After the 2-day recovery period, the hepatocytes were treated with SCO-201 or a known inducer (Table S3) in the incubation medium on day 3 and day 4. The known inducer was tested as the positive control. On day 5, the medium was removed, and the cells were incubated with the respective CYP substrate in Krebs–Henseleit buffer (pH 7.4) containing 3 mM salicylamide for 30 min. The reaction was terminated by transferring the incubation mixture to an equal volume of acetonitrile/methanol mixture (1/1, *v*/*v*). Samples were mixed and centrifuged. Supernatants are used for HPLC-MS/MS analysis of the corresponding metabolite. Peak areas corresponding to the metabolite were recorded. The assay was rendered valid if enzyme activity with the positive control was at least twofold greater than negative control.

### 2.12. Statistical Analyses 

Statistical analyses were performed using Microsoft Excel. Means and standard deviations were calculated for all quantitative data. For data represented in percentage (i.e., cell viability), the standard deviations, determined from triplicate experiments, were calculated and displayed on the graphs as standard deviation percentages: $Stdv\%=Stdv*\left(\frac{\%ofcontrol}{ODaverage}\right)$. Two-tailed, type 3 Student’s *t*-tests were applied on datasets where relevant, in order to determine any significant statistical differences. Statistical analysis of the data in the bidirectional transport assay was performed by comparing group means with a Student’s *t*-test (two-tailed) or ANOVA, followed by Bonferroni’s multiple comparisons test. The significance level was set to 5%, and thus *p*-values less than 0.05 were considered significant. 

## 3. Results

### 3.1. SCO-201 Reversed BCRP-mediated Drug Resistance in Chemotherapy Resistant Cells

**Figure 2:**
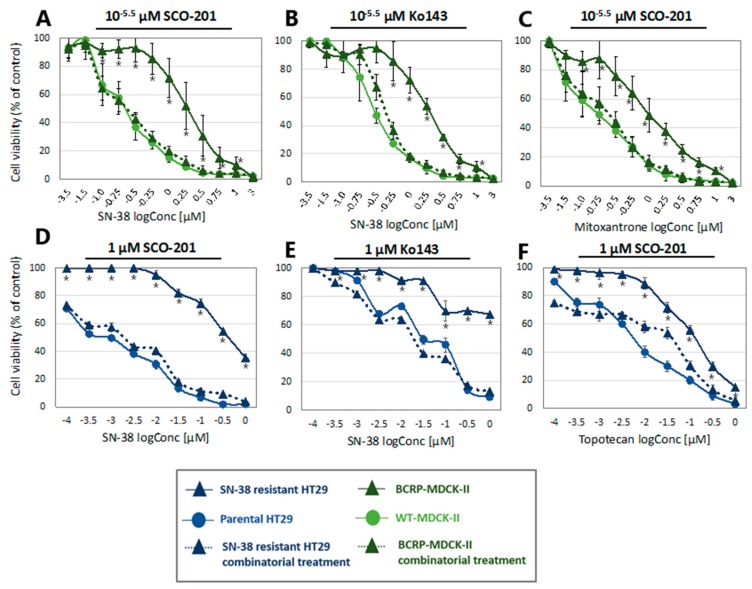
SCO-201 reversed drug resistance in multidrug resistance (MDR) cells. Cell viability of wild-type or breast cancer resistance protein (BCRP)-overexpressing MDCK-II cells and SN-38-sensitive or -resistant HT29 cells. Cell viability is indicated as percentage of untreated control, and error bars indicate percentage SD determined on the basis of *n* = 3–4. Note that some error bars might be invisible due to the size of the data labels. Control conditions consisted of full growth medium. (**A**–**C**) Wild-type and BCRP-overexpressing MDCK-II cells treated with either chemotherapy alone or in combination with 10^−5.5^ µM SCO-201 or Ko143. (**D**–**F**) Parental and SN-38-resistant HT29 cells treated with either chemotherapy alone or in combination with 1 µM SCO-201 or Ko143. Statistical difference (*p* < 0.05) between mono-treatment and combination treatment of the resistant cell lines is marked by * (Student’s *t*-test (two-tailed, type 3)).

To assess the potential BCRP-dependent re-sensitizing effects of SCO-201, we used the BCRP- transduced canine kidney subline, MDCK-II-BCRP, which in several studies has been shown to express high levels of BCRP [37,44]. The MDCK-II-BCRP cells are known to be less sensitive to chemotherapy substrates of BCRP, such as SN-38, topotecan, and mitoxantrone, compared to their parental counterpart, MDCK-II-WT [37,44]. We therefore tested the potential of SCO-201 to restore the drug sensitivity of the MDCK-II-BCRP cells to SN-38 and mitoxantrone by treating the cells with either chemotherapy alone or in combination with SCO-201, or the BCRP inhibitor Ko143 for comparison. As seen in Figure 2A-C, SCO-201 was able to completely restore the response to SN-38 and mitoxantrone in the MDCK-II-BCRP cells similar to the BCRP-inhibitor Ko143. The IC_50_ values are shown in Table 1. Treatment with SCO-201 and SN-38 or mitoxantrone resulted in decreased IC_50_ values for the MDCK-II-BCRP cells that were comparable to the IC_50_ values found for the MDCK-II-WT cells. Altogether, these results show that SCO-201 significantly re-sensitized MDCK-II-BCRP cells to both SN-38 and mitoxantrone. This is proof-of-concept that SCO-201 can affect BCRP-mediated resistance in a model system where the resistance is engineered by ectopic overexpression of BCRP.

To further investigate the re-sensitizing effects of SCO-201 and to apply a more complex model system of resistance, we tested the effects of SCO-201 in our DEN-50R in vitro model system of acquired SN-38 resistance in colorectal cancer. To generate this system, the colorectal adenocarcinoma cell line, HT29, was subjected to gradually increasing SN-38 concentrations for a period of ≈10 months, resulting in an SN-38-resistant cell line (HT29_SN-38-RES_). Genome-wide expression mRNA profiling revealed that BCRP was highly upregulated (25-fold) in the HT29_SN-38-RES_ cells, compared to their parental counterpart (HT29_PAR_) (GEO—Gene Expression Omnibus, NCBI, accession number GSE42387) [25]. We confirmed the BCRP overexpression with Western blot analysis (Figure S1). As seen in the blot, two bands could be observed for BCRP in the HT29_SN-38-RES_ cells, most likely due to the glycosylation states of BCRP [45]. 

We tested the potential re-sensitizing effects of SCO-201 in the HT29_SN-38-RES_ cells by treating the cells with either chemotherapy alone or in combination with SCO-201 or Ko143. As seen in Figure 2D–F, SCO-201 was able to significantly restore the response to both SN-38 and topotecan in the HT29_SN-38-RES_ cells, similar to the response observed for combinatorial treatment with Ko143. The IC_50_ values are shown in Table 1. The IC_50_ values of SN-38 and topotecan decreased several folds for the HT29_SN-38-RES_ cells, following treatment with SCO-201, and they almost completely reached the IC_50_ values for the HT29_PAR_ cells (Table 1).

To investigate if these observations were due to general damaging effects on the cells that could lead to general increase in sensitivity to chemotherapy, we applied oxaliplatin, which is not a substrate for BCRP. When oxaliplatin was combined with either SCO-201 or Ko143 in the HT29_SN-38-RES_ cells, no added effects were observed (Figure S2). This suggests that the re-sensitizing effects were not due to general cellular effects of either SCO-201 or Ko143. 

### 3.2. SCO-201 Inhibited the BCRP-Mediated Flux Across Cell Membranes 

**Figure 3:**
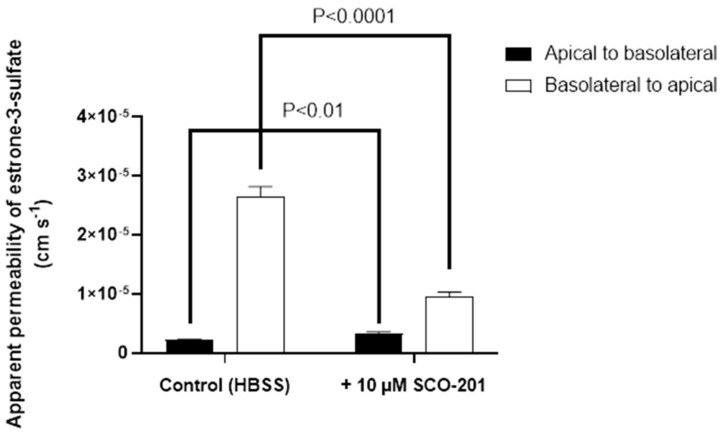
Bidirectional transport of estrone-3-sulfate across Caco-2 cell monolayers in the presence or absence of 10 µM SCO-201. Permeability (Papp) values were calculated from steady-state fluxes as described the in the Methods section (Section 2.5). Filled bars show PA-B (apical to basolateral) values, open bars show PB-A (basolateral to apical) values. Values are means ± SD of three individual passages, with three individual permeable supports for each transport direction per passage (*n* = 3−5, total *N* = 9). The *p*-values are indicated in the figure.

To more directly investigate whether SCO-201 inhibits BCRP-mediated efflux transport, a series of bidirectional transport experiments with the prototypical BCRP substrate [^3^H]-estrone-3-sulfate were completed across monolayers of Caco-2 cells (Figure 3). In the absence of SCO-201, the apparent permeability of [^3^H]-estrone-3-sulfate in the efflux direction (basolateral to apical) was 2.7 ± 0.2 × 10^−5^ cm/second, whereas it was considerably lower in the opposite direction with an apparent permeability of 2.2 ± 0.01 × 10^−6^ cm/second. The resulting efflux ratio in the absence of SCO-201 was 12.2, which indicated a marked polarized transport in the efflux direction for [^3^H]-estrone-3-sulfate across monolayers of Caco-2 cells. In the presence of 10 µM SCO-201, the apparent B-A (basolateral to apical) permeability was significantly reduced to 9.6 ± 0.7 × 10^−6^ cm/second (*p* < 0.0001), whereas the apparent permeability in the A-B (apical to basolateral) direction was significantly increased to 3.2 ± 0.3 × 10^−6^ cm/second (*p* = 0.0061). Correspondingly, the calculated efflux ratio was markedly reduced to 2.9, which together with the reduction in efflux transport of [^3^H]-estrone-3-sulfate were clear indications that SCO-201 had an inhibitory effect on BCRP-mediated efflux transport of [^3^H]-estrone-3-sulfate across Caco-2 cell monolayers (Figure 3).

**Figure 4:**
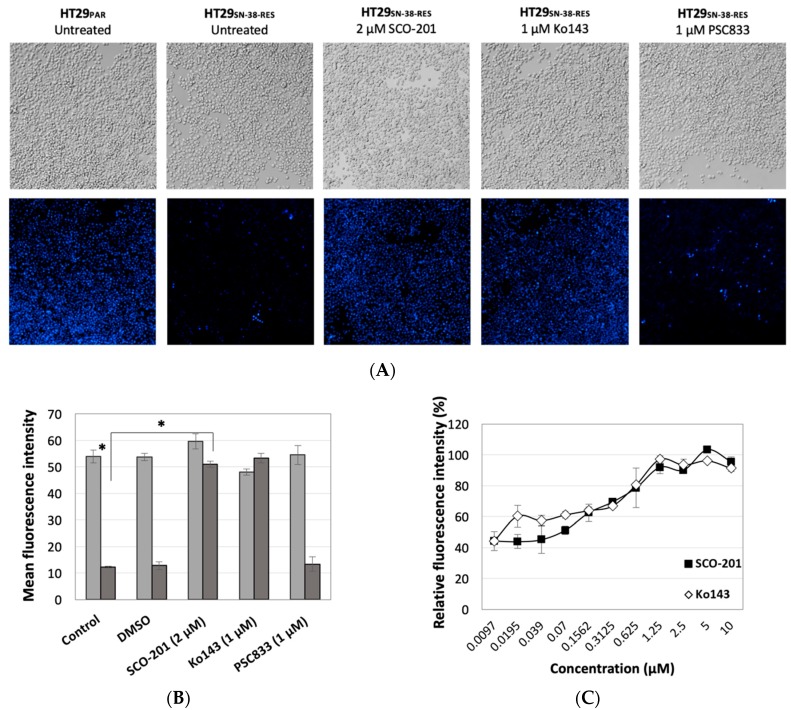
SCO-201 inhibited the efflux of Hoechst 33,342 from HT29_SN-38-RES_ cells. (**A**) Fluorescence micrographs of Hoechst-stained parental HT29 cells (HT29_PAR_) and HT29_SN-38-RES_ cells that were incubated with either SCO-201, the BCRP-inhibitor Ko143, or the MDR1-inhibitor valspodar (PSC833)**.** Full growth medium was used for the control condition. Untreated parental HT29 cells were included as a positive control, indicating the maximal accumulation of Hoechst dye, as these cells do not overexpress BCRP. (**B**) Mean fluorescence intensity of Hoechst-stained SN-38-sensitive (light grey) and SN-38 resistant (dark grey) HT29 cells treated with either DMSO, SCO-201, Ko143, or PSC833. Full growth medium was used for the control condition. The asterisks (*) indicate statistical significance (*p* < 0.05). **(C)** The dose-dependent effects of SCO-201 and Ko143 on the accumulation of Hoechst, indicated by the increase in relative fluorescence intensity of Hoechst-stained HT29_SN-38-RES_ cells. Error bars in (**B**,**C**) indicate SD determined from triplicate experiments.

To further elucidate whether SCO-201 modulates BCRP and in this way triggers intracellular accumulation of chemotherapy, we conducted dye efflux studies on wild-type and SN-38-resistant HT29 cells, the latter of which overexpresses BCRP. The cells were stained with Hoechst in the presence or absence of SCO-201, Ko143, or the MDR1/P-gp inhibitor PSC833 as a negative control. Figure 4 presents results from fluorescence microscopy and imaging cytometry analysis. Accumulation of Hoechst could clearly be detected in the parental cells, whereas only low levels of Hoechst accumulation could be detected in the BCRP-overexpressing chemotherapy-resistant cells (Figure 4A,B). When the resistant cells were treated with either SCO-201 or Ko143, Hoechst accumulated to the level of the parental control cells, whereas treatment with PSC833 did not have any effects (Figure 4A,B). In a similar experiment, we quantified the dose-dependent effects of SCO-201 compared with Ko143 and evaluated the outcome with imaging cytometry. Figure 4C shows the dose-dependent effect of SCO-201 on intracellular accumulation of dye in Hoechst-stained cells compared to Ko143. As seen from the IC_50_ values, the potency of Ko143 (IC_50_ = 0.37 µM) and SCO-201 (IC_50_ = 0.45 µM) was almost identical. Altogether, these results indicate that SCO-201, like Ko143, modulates the efflux of dye via BCRP, resulting in an accumulation of Hoechst dye in the HT29_SN-38-RES_ cells. The same tendency could be observed when we quantified the effect of SCO-201 on the accumulation of Hoechst or Pheophorbic A in the MDCK-II-BCRP cells (Figure S5 and S6). These Supplementary studies also indicated that SCO-201 is likely not an inhibitor of MDR1/P-gp.

### 3.3. The Drug-Stimulated ATPase Activity of BCRP Was Competitively Inhibited by SCO-201

**Figure 5:**
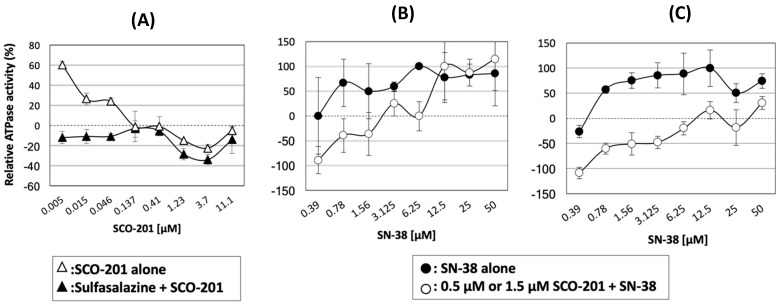
SCO-201 inhibited drug-stimulated ATPase activity of BCRP. (**A**) The effect of increasing concentrations of SCO-201 on basal and sulfasalazine-stimulated ATPase activity of BCRP. The data are indicated as relative ATPase activity, normalized with respect to basal (untreated) and maximal (sulfasalazine-stimulated) ATPase activity of BCRP. Sulfasalazine alone resulted in a maximal ATPase activity of 104.8 nmol Pi/mg protein/min compared to the baseline activity of 50.68 nmol Pi/mg protein/min. (**B**,**C**) The effect of 0.5 µM (B) and 1.5 µM (C) SCO-201 on SN-38-stimulated ATPase activity of BCRP. Error bars on all graphs represent SD determined from duplicates.

Following our results from the flux studies (Figure 3 and Figure 4), we further evaluated whether SCO-201 indeed is a modulator of BCRP transport activity. To evaluate the modulatory effects of SCO-201, we conducted ATPase studies to test the effect of SCO-201 on the ATPase activity of BCRP in the presence or absence of another activating BCRP substrate. Figure 5A shows the relative ATPase activities of BCRP incubated with either SCO-201 alone, or in the presence of the strong BCRP activator, sulfasalazine. Sulfasalazine alone resulted in a maximal ATPase activity of 104.8 nmol Pi/mg protein/min compared to the basal activity of 50.68 nmol Pi/mg protein/min (data not shown). As seen on Figure 5A, SCO-201 was not in itself a stimulator of BCRP ATPase activity, and in the absence of sulfasalazine, the ATPase activity of BCRP decreased dose-dependently upon incubation with SCO-201. When SCO-201 was added to sulfasalazine-stimulated BCRP, the ATPase activity of BCRP decreased in a dose-dependent manner (Figure 5A). Then, we tested the effect of two different concentrations of SCO-201 on SN-38-stimulated BCRP. Figure 5B,C shows the relative SN-38-stimulated ATPase activity of BCRP in the presence or absence of either 0.5 µM or 1.5 µM SCO-201. In the presence of SCO-201, the SN-38-stimulated ATPase activity of BCRP was shifted downwards, and the higher the SCO-201 concentration, the higher the SN-38 concentration was needed to reach the maximal activity of SN-38-stimulated BCRP. This revealed a typical competitive inhibition mechanism, showing that SCO-201 competed for the drug binding to the active site of BCRP. Altogether our data indicate that SCO-201 competitively inhibits the drug-stimulated ATPase activity of BCRP, suggesting that SCO-201 is a direct modulator of BCRP. 

### 3.4. Molecular Binding Model Further Supported a Competitive Action of SCO-201

**Figure 6:**
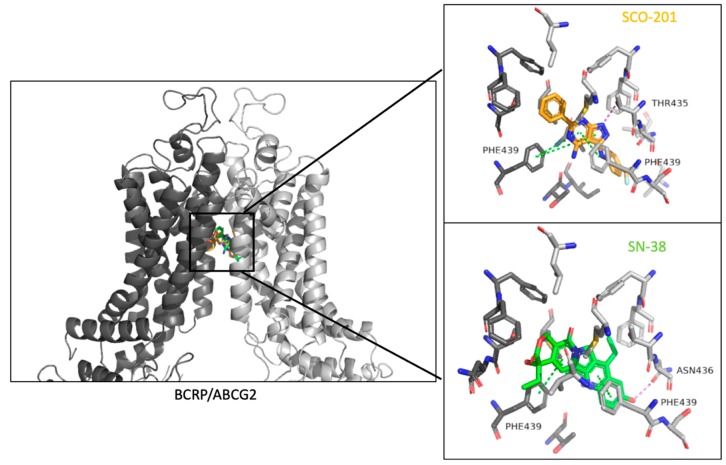
Docking of SN-38 and SCO-201 in the human BCRP transporter. The transporter is a homomeric dimer with chain A (dark grey) and chain B (light grey). SN-38 (green) and SCO-201 (orange) bind in the same binding cavity when docked using Glide, Schrödinger, 2019-3, LLC [39,40,41]. Both the substrate SN-38 and the proposed inhibitor SCO-201 interacted with Phenylalanine (PHE)-439 in both chains of the protein through hydrophobic Pi-stacking interactions. SCO-201 formed a hydrogen bond to Threonine (THR)-435, whereas SN-38 formed a hydrogen bond to Aspargine (ASN)-436. Pi-stacking interactions are colored green and hydrogen bonds are colored violet.

Thus far, our results have indicated that SCO-201 competitively inhibits the transport of BCRP substrates, such as SN-38, and that SCO-201 directly interacts with BCRP. To further support these results, we performed in silico molecular docking simulations to identify the binding sites of SCO-201 or SN-38 in BCRP. SN-38 and SCO-201 were successfully docked into the 3D structure of the BCRP and the resulting models showed that both SN-38 and SCO-201 were predicted to bind in the same binding cavity (Figure 6). Docking scores were -12.24 for SN-38 and -8.66 for SCO-201, indicating that both ligands could bind in the ligand binding site of the transporter. 

**Figure 7:**
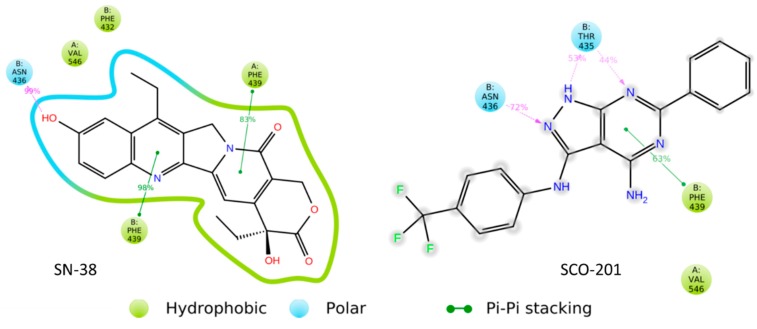
Binding interactions shown between SN-38 (left) and SCO-201 (right) to the transporter BCRP. Both molecules interacted with the PHE-439 residue of the B chain of BCRP. The interaction percentages indicate accounting for number of frames over the 10 ns simulation where the binding was present. Timetable of ligand–protein interactions are available in Figures S7 and S8. Interactions shown were present in over 30% of the simulation frames. Graphical representation was produced using Desmond, Schrödinger, 2019-3, LLC [43].

The molecular dynamic simulations demonstrated that both ligands remained in the binding sites throughout the sampled time period (Videos S1 and S2). The simulation interaction diagram (Figure 7) showed that SN-38 interacted through pi-stacking interactions with Phenylalanine (PHE)-439 of both protein chains, whereas SCO-201 predominantly interacted with the PHE-439 residue of the B chain and only to a lesser extent with the residue of the A protein chain (Figure 7; Figures S7 and S8). SN-38 further formed a hydrogen bond to Aspargine (ASN)-436 residue of the B chain of the transporter, whereas SCO-201 formed a hydrogen bond to both the Threonine (THR)-435 and ASN-436 residues of the B chain (Figure 7). As SCO-201 binds in the same binding cavity and interacts with some of the residues that the substrate SN-38 interacts with, it is likely that SCO-201 is a competitive inhibitor of the BCRP transporter by blocking substrate access. 

### 3.5. In Vitro DMPK Data Suggest That SCO-201 Is Not Likely to Increase The Risk of Pharmacokinetic Interactions

In clinical trials with ABC transporter inhibitors, pharmacokinetic interactions gave rise to increased serum levels of chemotherapy, thus enhancing the toxic effects of the chemotherapy, and dose reductions were therefore needed [34,35,36,37,38]. These dose reductions resulted in patients being under- or even overdosed, as the pharmacokinetic profile of each individual patient was difficult to predict [46,47,48,49,50]. On the basis of this, we aimed to test the potential inhibitory effects of SCO-201 on common transporters, playing a key role in pharmacokinetics, in order to predict the possibility that SCO-201 would negatively influence the pharmacokinetic profile of co-administered drugs. Specifically, we tested the inhibitory effects of SCO-201 on MDR1/P-gp as well as on several members of the solute carrier (SLC) family involved in drug pharmacokinetics, in accordance with the European Medicines Agency (EMA) Guidance of regulatory requirements for toxicological assessment of small molecules.

MDR1/P-gp and members of the SLC family including the organic anion transporting polypeptides (OATP) OATP1B1, OATP1B3, organic anion transporter (OAT) OAT1 and organic cation transporters (OCT) OCT2 act as major determinants of the absorption, distribution, excretion, and toxicity (ADME-tox) properties of drugs [51,52]. To investigate if SCO-201 inhibits any of the aforementioned transporters, we conducted a cell-based fluorometric drug transporter inhibition assay. We included BCRP as a positive control. The results are presented in Table 2 and show that SCO-201 inhibited BCRP as expected but is not likely an inhibitor of either MDR1/P-gp or any of the tested SLC family members as none of the fluorometric substrates accumulated. In addition, it is seen that the IC_50_ value for BCRP in CHO cells was 1.7 µM, showing that SCO-201 is a potent inhibitor of BCRP (Table 2 and Figure S7).

To further predict any potential risk of drug–drug interactions, we tested the effect of SCO-201 on key oxidative metabolic drug enzymes of the cytochrome P450 (CYP) family. Specifically, we tested the potential inhibition or induction by SCO-201 on a range of common CYPs playing a key role in determining the pharmacokinetic profile of drugs [53]. We used a standard CYP inhibition assay based on human liver microsomes, and the IC_50_ values of SCO-201 towards CYP1A, CYP2B6, CYP2C8, CYP2C9, CYP2C19, CYP2D6, and CYP3A (with two substrates) were determined in a range of concentrations (from 0.03 to 100 µM). The results are shown in Table 3 and found that no IC_50_ value was less than 100 µM, suggesting that SCO-201 is not likely an inhibitor of these CYP isoforms and may not cause CYP inhibition when its plasma concentration is below 100 µM. 

Inductions of CYP1A2, CYP2B6, and CYP3A4 by SCO-201 were tested at 1, 10, and 100 µM using both enzyme activity and mRNA level changes as the end-points. The results are presented in Table 4. Using enzyme activity as the end-point, the results were all below the cutoff value (40% of positive control), suggesting that SCO-201 is not an inducer for these CYP isoforms. Using mRNA level as the end-point, the results were also below the cutoff values, similarly suggesting that SCO-201 does not induce the CYP isoforms. However, there was a trend in both enzyme and mRNA assays that fold induction decreased with the increase in test concentrations. This trend may have resulted from cytotoxicity toward the hepatocytes. 

In conclusion, our results from these in vitro analyses might imply that SCO-201 does not significantly negatively influence the pharmacokinetic profile of co-administered drugs. This suggest that SCO-201 may be of a new generation of ABC transporter modulators with low risk of increased chemotherapy-mediated toxicity.

## 4. Discussion

ABC transporter-mediated resistance to multiple anti-cancer drugs is one of the major reasons for cancer treatment failure [1,2,3,4,5]. Overexpression of the transporter BCRP prevents chemotherapeutic agents such as SN-38, mitoxantrone, and fluoruracil from remaining inside cancer cells, and in this way, protects the cancer cells from being killed by these drugs. BCRP expression in cancer cells confers drug resistance in leukemia, and higher levels are reported in solid tumors from the digestive tract, endometrium, lung, and melanoma, although, contrarily, expression is generally low in breast cancer tumours [54]. There is significant association between BCRP expression and tumor response to chemotherapy and progression-free survival [1,2,3,4].

In this study, we showed that the pyrazolo-pyrimidine derivative SCO-201 can reverse MDR in vitro by competitively inhibiting the transport function of BCRP. Firstly, we tested the ability of SCO-201 to re-sensitize drug-resistant MDCK-II-BCRP and HT29_SN-38-RES_ cells to chemotherapy, and these data demonstrated that SCO-201 can successfully reverse resistance in these cells (Figure 2). To investigate the potential mechanism of action of SCO-201, we conducted dye efflux assay and examined the intracellular accumulation of Hoechst 33,342 in BCRP-overexpressing cells, when treated with SCO-201, by fluorescence microscopy and imaging cytometry (Figure 4). Our results indicated that SCO-201 triggers the accumulation of Hoechst 33,342 in the BCRP-expressing cells, similarly to Ko143. We subsequently performed ATPase assay in order to examine if the effect of SCO-201 was caused by a direct inhibition of the transport function of BCRP. These results showed that SCO-201 competitively inhibits the drug-stimulated activity of BCRP (Figure 5).

To further support these results, we performed molecular docking and molecular dynamics simulations, and found that SCO-201 and SN-38 were predicted to bind in the same binding pocket of BCRP. Both SN-38 and SCO-201 interacted with PHE-439 of the B protein chain through Pi-stacking hydrophobic interactions, and ASN-436 through hydrogen bonds (Figure 6 and Figure 7). From the cryo-EM structure of BCRP (PDB ID: 6ETI), stacking interaction was also seen between the inhibitor and PHE-439, which emphasized the importance of these residues both in transport and in inhibition of BCRP. It further supports SCO-201 being a competitive inhibitor of the BCRP transporter [16].

There are challenges of bringing ABC transporter inhibitors to clinical use, reflected by the fact that after 40 years of research there are still no approved ABC transporter inhibitors for use in the clinical setting [1]. To date, three generations of different MDR1/P-gp inhibitors have been tested and developed pre-clinically and clinically [1,7,9]. The first generation of inhibitors tested in clinical trials were not specifically developed to modulate ABC transporters but were drugs already in clinical use (e.g., verapamil and cyclosporine A). These were weak inhibitors and needed high doses. Such high doses in combination with anti-cancer drugs (e.g., mitoxantrone, daunorubicin, and etoposide) caused toxic side effects and had low therapeutic response [7,8,9,10]. Therefore, these were quickly replaced by more potent and specific second-generation inhibitors (e.g., R-verapamil and PSC-833 (Valspodar)) in order to reduce possible primary toxicities. In acute myeloid leukemia (AML) patients, the combination of PSC-833 with anti-cancer drugs seemed to be beneficial for some patients. However, the second-generation inhibitors were also shown to be inhibitors of CYPs and displayed pharmacokinetic interactions leading to increased toxicity [7,8,9,10]. Several phase III clinical studies with PSC-833 revealed that the combination with chemotherapeutic agents did not prolong the survival of cancer patients [47,48,50,55]. Third-generation inhibitors (e.g., laniquidar (R101933), ONT-093 (OC14–093), zosuqiodar (LY335979), elacridar (GF120918), and tariquidar (XR9576)) were up to 200-fold more potent and had low pharmacokinetic interaction due to a limited CYP3A inhibition [56]. The third-generation inhibitors are well tolerated in humans, safe to combine with chemotherapy due to less systemic pharmacokinetic interactions than previous MDR1 inhibitors, and were found to cause potent MDR1/P-gp inhibition in humans [57,58,59,60,61,62,63]. Furthermore, scanning imaging of tumors for contents of (99m)Tc-sestamibi before and after dosing of third generation inhibitors could possibly be applied to identify subgroups of anti-cancer-resistant cancer patients who may benefit from a combination of inhibitor and anti-cancer drug. 

Thus, the development from first to third generation inhibitors has to a large degree abolished the pharmacokinetic interactions and related toxicities with the tested inhibitors and anti-cancer drugs. Even if toxicities should be observed with novel inhibitors and combinations with anti-cancer drugs, this can be taken care of by starting a patient with an ABC transporter inhibitor and reduced dose of chemotherapy. If no severe side effects are noted, the dose of chemotherapy can be increased at the next cycle. It is important to remember that, even with a need for lowering the dose of chemotherapy, a significant anti-tumor effect might be obtained due to the simultaneous inhibition of drug efflux pumps in the cancer cells. Another problem with the clinical studies, which tested the efficacy of ABC transporter inhibitors in combination with chemotherapy, was the general lack of randomization, and in most studies the ABC transporter inhibitor was not combined with the chemotherapy that the patient had developed resistance against. Finally, the clinical studies also lacked the inclusion of predictive biomarkers. In some of the studies, a few of the patients had ABC cassette proteins measured in their tumor tissue, but in none of the studies was this performed on a fresh tumor biopsy, and was instead performed on the primary biopsy obtained at the time of diagnosis and prior to any chemotherapy, which does not necessarily reflect the expression of ABC transporter proteins in the resistant tumor cells.

To our knowledge, no second or third generation BCRP inhibitors have been developed and tested in clinical studies. The BCRP inhibitors tested so far are first generation inhibitors, which are developed to inhibit other targets, and have pharmacokinetics interactions [64]. Some of the mechanisms by which ABC transporter inhibitors could alter the pharmacokinetics of the anti-cancer agent include competition for CYPs, intestinal or liver metabolism, inhibition of ABC transporter-mediated biliary excretion or intestinal transport, or inhibition of renal excretion and elimination [65]. This means that for an inhibitor to succeed, it needs to be non-toxic itself, and have no or low risk of interaction with important pharmacokinetic proteins, such as CYPs.

In this study, in vitro DMPK analyses of SCO-201 demonstrated no inhibition or induction of CYPs or SLCs whatsoever, suggesting a reduced risk of drug–drug interactions with other drugs, such as chemotherapy (Table 2, Table 3 and Table 4). As mentioned, an obstacle for inhibitors to succeed in clinical development is the fact that these also inhibit ABC transporters found in healthy tissues, which may lead to increased toxic effects of chemotherapy. This is especially a problem with broad-spectrum inhibitors that interact with many different efflux and uptake transporters, such as other ABC transporters or SLCs. However, by applying a specific inhibitor, this will allow the other ABC transporters in healthy tissues to compensate for the inhibition of the specific transporter, thereby protecting the healthy tissue from the toxic effects of the chemotherapy. In contrast, the anti-cancer drug has induced an up-regulation of the specific transporter in the resistant cancer cells, which will therefore be re-sensitized to the toxic effects of the anti-cancer drug. Therefore, it is highly likely that the more specific the inhibitor, the lower is the risk of increased toxicity. The specificity of SCO-201 to BCRP could provide benefits to the safety and tolerability profile in co-medication in cancer treatment compared to application of broad-spectrum inhibitors. Thereby, a BCRP-specific inhibition reduces the risk of potential drug–drug interactions in co-medication, thus decreasing the risk for increased chemotherapy-mediated toxicity.

To our best knowledge, a specific BCRP inhibitor has never been in clinical testing. Future in vivo studies of SCO-201 in combination with BCRP chemotherapy substrates should be conducted to further examine the pharmacology and test for potential increased chemotherapy-mediated toxicity. In such studies, we should utilize all the prior knowledge obtained with ABC transporter inhibitors. This means that we should carefully select patients with acquired drug resistance (a prior benefit to the chemotherapy in question), test for biomarkers such as cancer cell ABCG2 expression, start with a reduced dose of chemotherapy (the chemotherapy that the patient had acquired resistance against, and it should be an ABCG2 substrate drug) in combination with the inhibitor, perform randomization of patients in order to include time-dependent end-points such as progression free survival and overall survival, and perform a post-treatment association study between patient outcome and biomarkers. In vivo pharmacokinetic studies of other pyrazolo-pyrimidine derivatives, such as Reversan [19,34], have indicated that these do not cause increased toxicity of chemotherapy, and therefore it is possible that SCO-201 also will not cause these unwanted toxic effects in vivo.

## 5. Conclusions

Altogether, our data suggest that SCO-201 is a potential new drug candidate for the reversal of BCRP-mediated resistance in cancer. SCO-201 appears to be a specific and potent inhibitor of BCRP without affecting the CYP450 levels. Additionally, SCO-201 is stable in serum, has a favorable pharmacokinetic, toxicological, and pharmacodynamic profile in mice, and is orally active [33]. We conclude that SCO-201 is a highly promising drug candidate for drug-resistant cancer where overexpression of BCRP is the key mechanism of drug resistance. 

## Figures and Tables

**Table 1:** IC_50_ values of anti-cancer drugs in the presence/absence of SCO-201 or Ko143.

	IC_50_ Value (µM) for Each Cell Line			
Drug/Drug Combination	MDCK-II-WT	MDCK-II-BCRP	HT29_PAR_	HT29_SN-38-RES_
SN-38	0.214 ± 0.023	1.956 ± 0.080	0.016 ± 0.010	0.463 ± 0.363
SN-38 + SCO-201		0.238 ± 0.029		0.007 ± 0.005
SN-38 + Ko143		0.437 ± 0.020		0.014 ± 0.021
Mitoxantrone	0.157 ± 0.023	1.060 ± 0.095		
Mitoxantrone + SCO-201		0.214 ± 0.030		
Topotecan			0.008 ± 0.003	0.121 ± 0.024
Topotecan + SCO-201				0.088 ± 0.030

Taken together, our results show that SCO-201 could successfully reverse resistance to the anti-cancer BCRP substrates, SN-38, topotecan, and mitoxantrone, similarly to the BCRP inhibitor Ko143 in BCRP over-expressing MDCK-II-BCRP and HT29_SN-38-RES_ cells. This indicates that SCO-201 could be a modulator of BCRP activity. Supplementary studies showed that SCO-201 has a dose-dependent effect with SN-38 in both MDCK-II-BCRP and HT29_SN-38-RES_ cells (Figures S3 and S4).

**Table 2:** Summary of drug transporter inhibition.

Transport Protein	Cell Line	Substrate	IC_50_ (M)
OCT2	OCT2-CHO	ASP+	NC
BCRP	BCRP-CHO	Hoechst 33342	1.7 × 10^−6^
OAT1	OAT-CHO	CF	NC
OAT3	OAT3-CHO	CF	NC
OATP1B1	OATP1B1-CHO	FMTX	NC
OATP1B3	OATP1B3-CHO	FMTX	NC
P-gp (MDR1)	MDR1-MDCK-II	Calcein-AM	NC

NC: not calculable.

**Table 3:** Cytochrome P450 (CYP) inhibition.

CYP	Test	Substrate	IC_50_ (M)
CYP1A	Human hepatocytes	Phenacetin substrate	>1 × 10^−4^
CYP2B6	Human hepatocytes	Bupropion substrate	NC
CYP2C8	Human liver microsomes	Paclitaxel	>1 × 10^−4^
CYP2C9	Human liver microsomes	Diclofenac	>1 × 10^−4^
CYP2C19	Human liver microsomes	Omeprazole	NC
CYP2D6	Human liver microsomes	Dextromethorphan	NC
CYP3A	Human liver microsomes	Midazolam	NC
		Testosterone	>1 × 10^−4^

NC: not calculable.

**Table 4:** CYP induction.

CYP	Mean Fold Induction of mRNA at 1× 10^−4^ M			Mean Fold Enzyme Activity Induction at 1 × 10^−4^ M		
	Donor 1	Donor 2	Donor 3	Donor 1	Donor 2	Donor 3
CYP1A				1	0.7	0.7
CYP1A2	0.24	0.39	0.26			
CYP2B6	0.17	0.52	0.45	0.8	0.9	0.6
CYP3A				1.3	2.0	0.4
CYP3A	0.25	0.25	0.32			

Fold induction = (activity of test compound treated cells)/(activity of negative control). mRNA fold induction = (mRNA level in test compound treated cells)/(mRNA levels in vehicle treated controls).

## Supplementary Materials

The following are available online at https://www.mdpi.com/2073-4409/9/3/613/s1. Figure S1: Western blot analysis of BCRP expression in SN-38-sensitive and SN-38-resistant HT29 cells. Figure S2: Cell viability assay with HT29 SN38-resistant colon cancer cells. Figure S3: Cell viability assay with mitoxantrone-resistant MDCK-II ATP-binding cassette (ABC)G2 cells. Figure S4: Cell viability assay with HT29 SN38-resistant colon cancer cells. Figure S5: Comparison of ABCG2 (BCRP) inhibition by SCO-201 and Ko143 as determined in the Hoechst 33,342 accumulation assay in MDCK cells. Figure S6: Comparison of SCO-201 and Ko143 as determined in the pheophorbide A accumulation assay in MDCK cells. Figure S7: SCO-201 (OBR-5-340)-mediated inhibition of BCRP. Figure S8: Timeline representation of interaction and contacts between residues in the binding cavity of the BCRP transporter and SCO-201. Figure S9: Timeline representation of interaction and contacts between residues in the binding cavity of the BCRP transporter and SN-38. Table S1: In vitro DMPK analysis of transporter inhibition. Table S2: In vitro DMPK analysis: CYP inhibition. Table S3: In vitro DMPK analysis: CYP induction.
