# Supplementary material for: The Pyrazolo[3,4-d]pyrimidine Derivative, SCO-201, Reverses Multidrug Resistance Mediated by ABCG2/BCRP
Source: Cells. 2020 Mar 4;9(3):613. doi: 10.3390/cells9030613 (PMC7140522; doi:10.3390/cells9030613)
Supplement: Supplementary file 1 [file cells-09-00613-s001.pdf]

Article

Supplementary Materials:
The Pyrazolo[3,4-d]pyrimidine Derivative, SCO-201, Reverses Multidrug Resistance Mediated by ABCG2/BCRP

Sophie E. B. Ambjørner[1], Michael Wiese[2], Sebastian Köhler[2], Joen Svindt[3], Xamuel Loft Lund[1], Michael Gajhede[1], Lasse Saaby[4], Birger Brodin[4], Steffen Rump[5], Henning Weigt[6], Nils Brünner[1,7], Jan Stenvang[1,7,*]

[1] Department of Drug Design and Pharmacology, University of Copenhagen, Copenhagen, Denmark; sophie.ambjoerner@gmail.com (S.A.); lxs184@alumni.ku.dk (X.L.L); mig@sund.ku.dk (M.G.)
[2] Pharmaceutical Institute, University of Bonn, Bonn, Germany; mwiese@uni-bonn.de (M.W.); skoehler@uni-bonn.de (S.K.)
[3] Department of Biology, University of Copenhagen, Copenhagen, Denmark; joen.svindt@gmail.com (J.S)
[4] Department of Pharmacy, University of Copenhagen, Copenhagen, Denmark; lasse.saaby@sund.ku.dk (L.S.); birger.brodin@sund.ku.dk (B.B.)
[5] SRConsulting, Sehnde, Germany; rump@s-r-consulting.com (S.R.)
[6] Division of Chemical Safety and Toxicity, Fraunhofer Institute of Toxicology and Experimental Medicine, Hannover, Germany; henning.weigt@item.fraunhofer.de (H.W.)
[7] Scandion Oncology A/S, Symbion, Copenhagen, Denmark; js@scandiononcology.com (J.S.); nb@scandiononcology.com (N.B.)
* Correspondence: js@scandiononcology.com; Tel: +45 60-777-678

Received: date; Accepted: date; Published: date

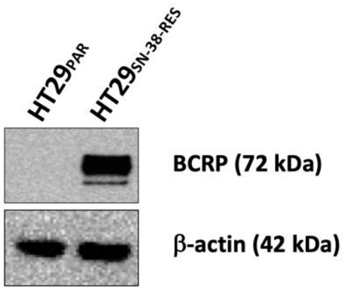

Figure S1: Western blot analysis of BCRP expression in SN-38 sensitive and SN-38 resistant HT29 cells. β-actin was used as loading control.

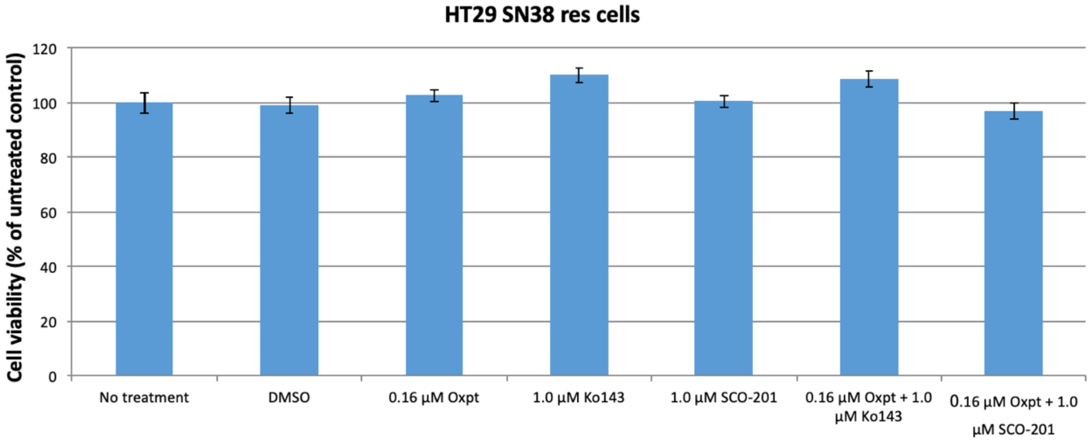

Figure S2: Cell viability assay with HT29 SN38 resistant colon cancer cells. Cells were incubated for 72 hours with the indicated concentrations of drugs. It is seen that the combination of neither SCO-201 or Ko143 with oxaliplatin (which is not a substrate for BCRP) has any combinatorial effect on the cell viability in the tested concentrations. A representative example is shown. Error bars indicate standard deviations.

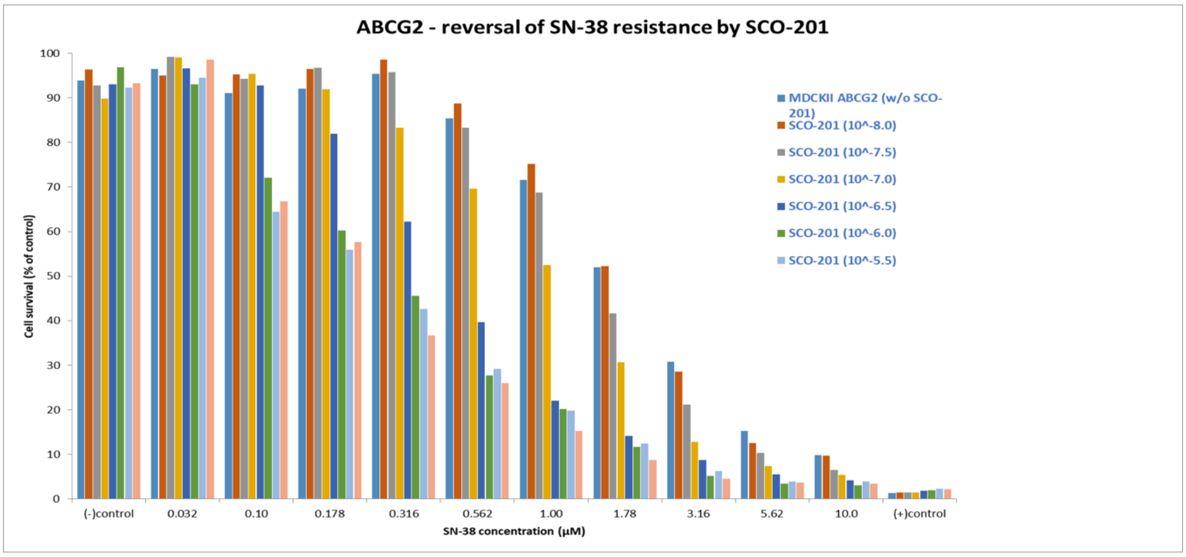

Figure S3: Cell viability assay with mitoxantrone-resistant MDCKII-ABCG2/BCRP cells. Cells were incubated for 72 hours with the indicated concentrations of drugs. It is seen that the cell viability is reduced in a dose-dependent manner by SN38 in dose-dependent manner.

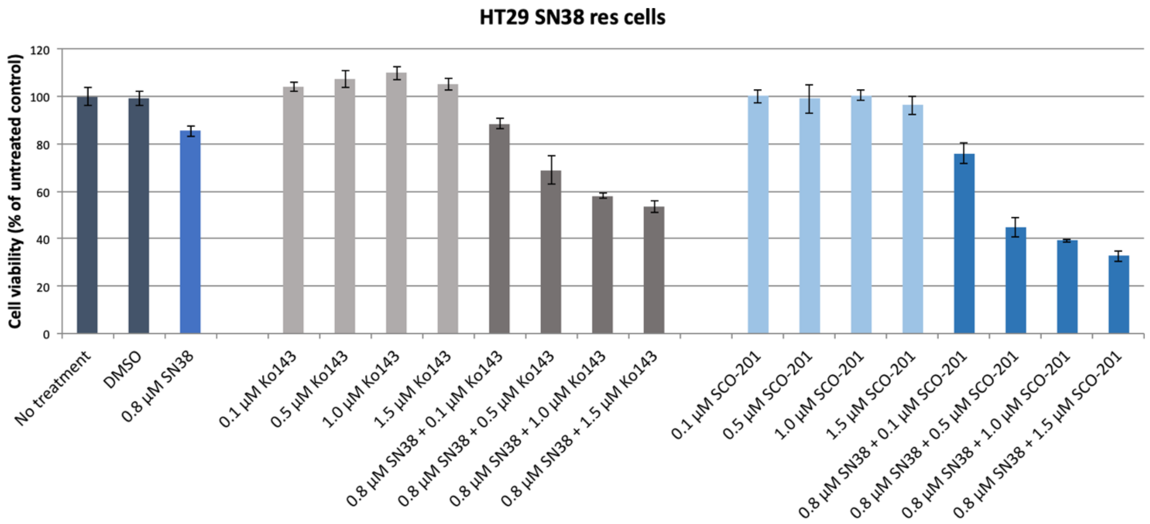

Figure S4: Cell viability assay with HT29 SN38 resistant colon cancer cells. Cells were incubated for 72 hours with the indicated concentrations of drugs. It is seen that the combination of both SCO-201 or Ko143 with SN38 has a combinatorial effect on the cell viability. It is also shown that SCO-201 is more potent than Ko14 in restoring the effect of SN38 in the SN38 resistant cells. A representative example is shown. Error bars indicate standard deviations.

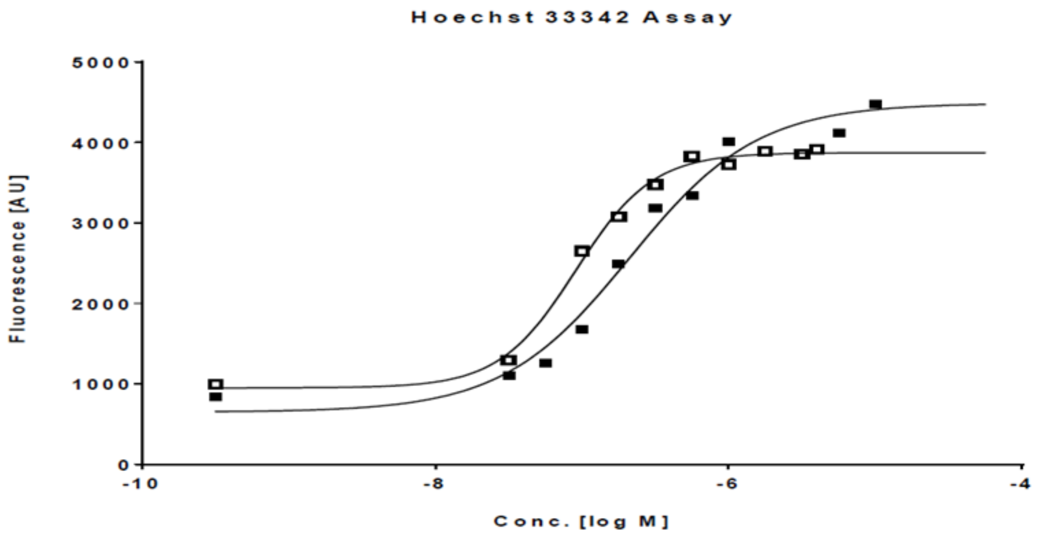

Figure S5: Comparison of ABCG2 (BCRP) inhibition by SCO-201 and Ko143 as determined in the Hoechst 33342 accumulation assay in MDCK cells. Open and closed squares indicate SCO-201 and Ko143, respectively. For comparative purposes, the potent and selective ABCG2 inhibitor Ko143, which is described in the literature as a standard inhibitor for potency assessment of new compounds, was used as a comparator. Different concentrations of SCO-201 and Ko143 in a logarithmic scale were tested and fluorescence values in the steady state measured for each concentration. The IC_{50}-values for SCO-201 and Ko143 were 0.175 (± 0.077) µM and 0.250 (± 0.106) µM, respectively. Thus, the inhibitory potential of SCO-201 on ABCG2 (BCRP) was higher compared to the standard inhibitors Ko143. A representative experiment is depicted.

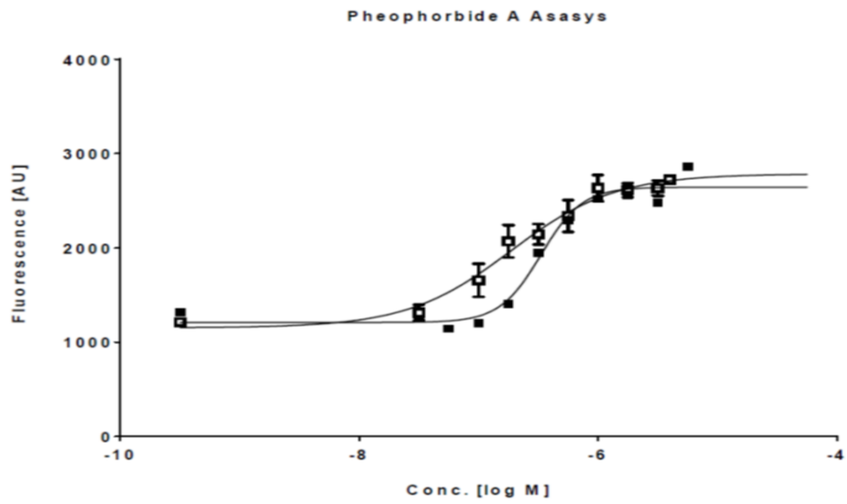

Figure S6: Comparison of SCO-201 and Ko143 as determined in the Pheophorbide A accumulation assay in MDCK cells. Open and closed squares indicate SCO-201 and Ko143, respectively. For comparative purposes, the potent and selective ABCG2 inhibitor Ko143, which is described in the literature as a standard inhibitor for potency assessment of new compounds, was used as a comparator. Different concentrations of SCO-201 and Ko143 in a logarithmic scale were tested and intra cellular fluorescence values in the steady state measured by flow cytometry for each concentration. IC_{50} value of SCO-201 in the pheophorbide A accumulation assay was determined for SCO-201 and Ko143 to 0.145 μM (±0.040 μM) and 0.231 (± 0.102) μM, respectively. Thus, the inhibitory potential of SCO-201 was higher compared to the standard inhibitors Ko143. A representative experiment is depicted.

Compound I.D.	Client Compound I.D.	IC_{50} (M)	nH	Test Concentration	% of Control Values		
					1st	2nd	Mean
**BCRP *(h)* inhibition (BCRP-CHO, Hoechst 33342 substrate)**							
100014197-1	OBR-5-340	1.7E-06 M	0.4	3.0E-08 M	94.2	95.2	94.7
				1.0E-07 M	83.7	84.9	84.3
				3.0E-07 M	64.8	63.3	64.1
				1.0E-06 M	45.3	42.3	43.8
				3.0E-06 M	39.3	41.4	40.4
				1.0E-05 M	30.0	31.8	30.9
				3.0E-05 M	26.5	25.9	26.2
				1.0E-04 M	27.4	23.5	25.4

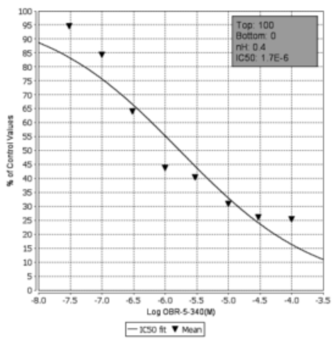

Figure X. OBR-5-340 on BCRP *(h)* inhibition (BCRP-CHO, Hoechst 33342 substrate)

Figure S7: SCO-201 (OBR-5-340) mediated inhibition of BCRP. A dye efflux assay employing the BCRP substrate Hoechst 33342 in BCRP overexpressing CHO cells determined the IC_{50} of SCO-201 to 1.7 μM.

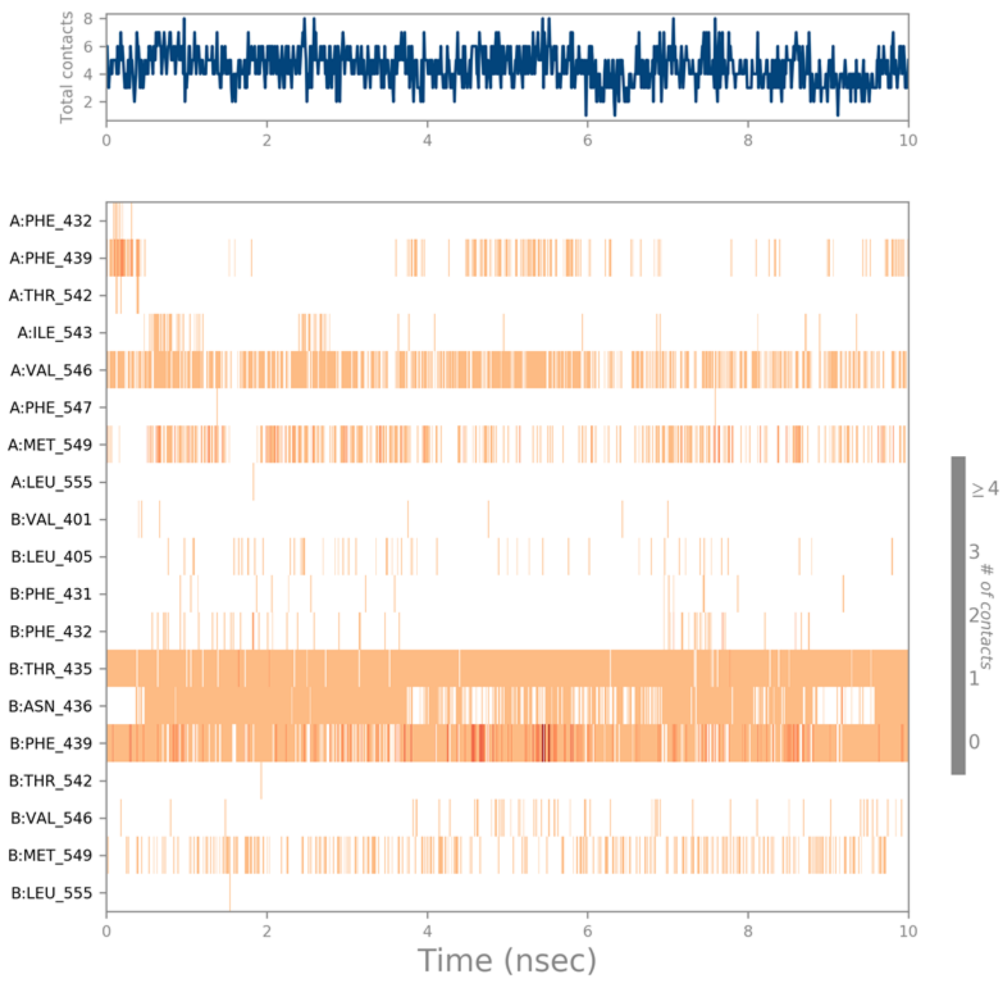

Figure S8: Figure S8: Timeline representation of interaction and contacts between residues in the binding cavity of the BCRP transporter and SCO-201. Interactions (hydrogen bonds, hydrophobic, ionic and water bridges) are shown over the simulation time. Residues which make more than one interaction or contact with the ligand are shown as a darker shade of orange. From the timeline it could be seen that SCO-201 interacts heavily with PHE-439 of the B chain. Further it shows that THR-435 and ASN-436 of the protein B chain interacts with SCO-201 and to a minor extent VAL-546 of the A chain of the transporter also interacts with the ligand through most of the simulation. Blue bar in the top of the figure shows the total number of interactions between ligand and transporter at a given time. Representation produced using Desmond, Schrödinger 2019-3, LLC [1].

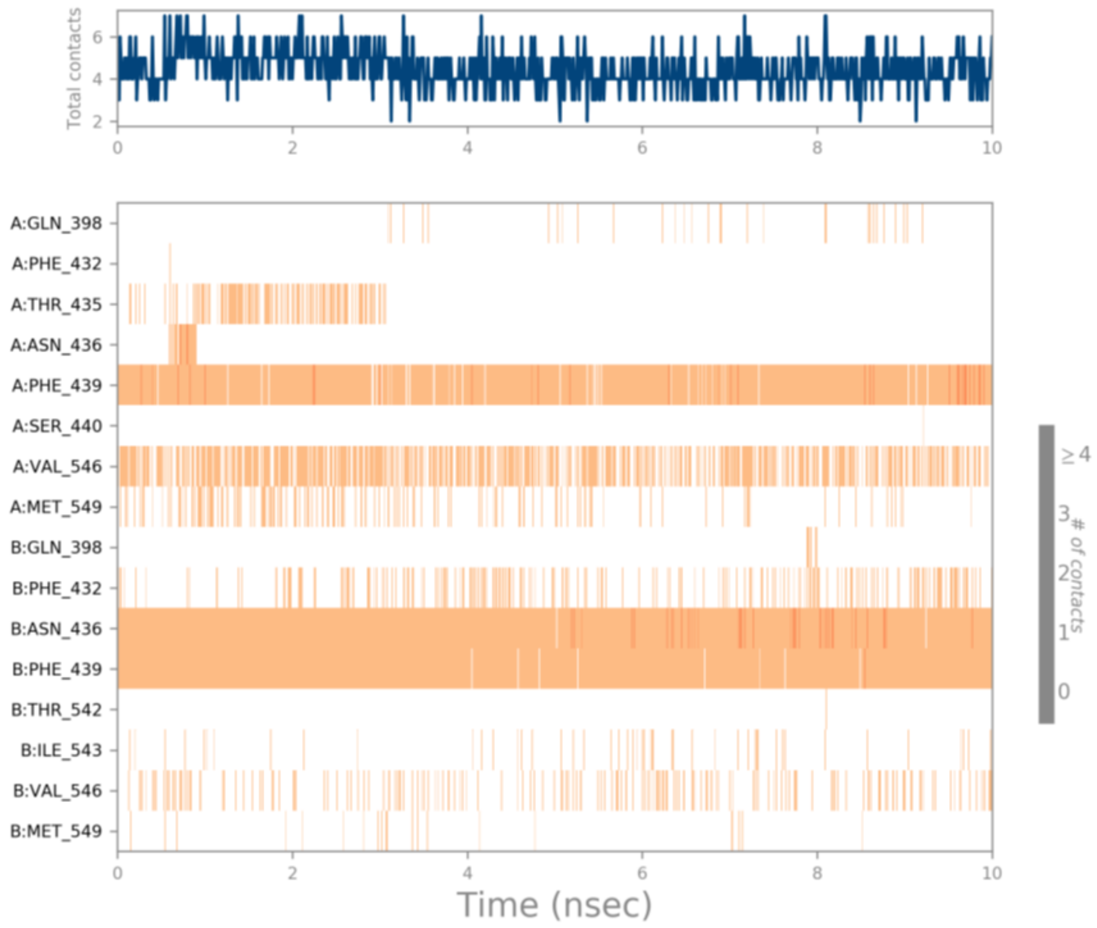

Figure S9: Timeline representation of interaction and contacts between residues in the binding cavity of the BCRP transporter and SN-38. Interactions (hydrogen bonds, hydrophobic, ionic and water bridges) are shown over the simulation time. Residues which make more than one interaction or contact with the ligand are shown as a darker shade of orange. From the timeline it could be seen that SN-38 interacts heavily with PHE-439 of both protein chains as well as ASN-436 from protein chain B. VAL-546 of the proteins chain A also interact with the substrate throughout most of the simulation. Blue bar in the top of the figure shows the total number of interactions between ligand and transporter at a given time.

Table S1. In vitro DMPK analysis of transporter inhibition

Transporter	Cell line	Substrate	Reference inhibitor
P-gp	MDR1-MDCKII	Calcein AM	Verapamil
BCRP	BCRP-CHO	Hoechst 33342	Ko143
OATP1B1	OATP1B1-CHO	Fluorescein methotrexate	Rifampicin
OATP1B3	OATP1B3-CHO	Fluorescein methotrexate	Rifampicin
OAT1	OAT1-CHO	6-carboxyfluorescein	Probenecid
OAT3	OAT3-CHO	6-carboxyfluorescein	Probenecid
OCT2	OCT2-CHO	ASP+	Verapamil

Reference Compound	IC_{50} (M)	nH
**OCT2 (h) inhibition (OCT2-CHO, ASP+ substrate)**		
verapamil	8.3E-06 M	0.5
**BCRP *(h)* inhibition (BCRP-CHO, Hoechst 33342 substrate)**		
KO143	5.5E-08 M	0.5
**OAT1 *(h)* inhibition (OAT1-CHO, CF substrate)**		
Probenecid	3.8E-05 M	0.8
**OAT3 *(h)* inhibition (OAT3-CHO, CF substrate)**		
Probenecid	1.9E-05 M	0.6
**OATP1B1 *(h)* inhibition (OATP1B1-CHO, FMTX substrate)**		
Rifampicin	2.2E-06 M	0.6
**OATP1B3 *(h)* inhibition (OATP1B3-CHO, FMTX substrate)**		
Rifampicin	5.4E-06 M	0.4
**P-gp inhibition (MDR1-MDCKII, calcein AM substrate)**		
Verapamil	3.1E-06 M	0.9

Table S1: In vitro DMPK analysis of transporter inhibition. As indicated in Table S1, several reference compounds were included in the DMPK transporter inhibition analyses to demonstrate the functionality of the transporter assay. The background for P-gp and BCRP is the mean reading in the presence of the highest effective concentration of the reference inhibitor. The background for OATP1B1, OATP1B3, OAT1, OAT3, and OCT2 is the mean reading in the absence of both the test compound and the substrate. For the efflux transporters (P-gp and BCRP), increased fluorescence signal represents inhibition of the transporter activity. For the uptake transporters (OATP1B1, OATP1B3, OAT1, OAT3, and OCT2), decreased fluorescence signal represented inhibition of the transporter activity. The IC50 values are shown for the analysed transporters with the indicated substrate.

Table S2. In vitro DMPK analysis: CYP inhibition

**Assay**	**Substrate**	**Metabolite**	**Reference inhibitor**
CYP1A	phenacetin	acetaminophen	furafylline
CYP2B6	bupropion	hydroxybupropion	clopidogrel
CYP2C8	paclitaxel	6α-hydroxypaclitaxel	montelukast
CYP2C9	diclofenac	4′-hydroxydiclofenac	sulfaphenazole
CYP2C19	omeprazole	5-hydroxyomeprazole	oxybutynin
CYP2D6	dextromethorphan	dextrorphan	quinidine
CYP3A	midazolam	1-hydroxymidazolam	Ketoconazole
CYP3A	testosterone	6β-hydroxytestosterone	Ketoconazole

**Reference Compound**	**IC_{50} (M)**	**nH**
**CYP1A inhibition (HLM, phenacetin substrate)**		
Furafylline	6.2E-06 M	0.9
**CYP2B6 inhibition (HLM, bupropion substrate)**		
Clopidogrel	7.4E-07 M	1.3
**CYP2C8 inhibition (HLM, paclitaxel substrate)**		
Montelukast	3.6E-06 M	0.8
**CYP2C9 inhibition (HLM, diclofenac substrate)**		
Sulfaphenazole	3.7E-07 M	0.8
**CYP2C19 inhibition (HLM, omeprazole substrate)**		
Oxybutynin	5.5E-06 M	1.0
**CYP2D6 inhibition (HLM, dextromethorphan substrate)**		
Quinidine	1.2E-07 M	1.1
**CYP3A inhibition (HLM, midazolam substrate)**		
Ketoconazole	2.1E-07 M	>3
**CYP3A inhibition (HLM, testosterone substrate)**		
Ketoconazole	2.7E-07 M	>3

Table S2: In vitro DMPK analysis: CYP inhibition. As indicated in Table S2, several reference compounds were included in the DMPK transporter inhibition analyses to demonstrate the functionality of the CYP inhibition assay. The IC_{50} values are shown for the analysed CYP's with the indicated inhibitors.

Table S3. In vitro DMPK analysis: CYP induction

**Positive control (conc.)**	**Assay**
Omeprazole (50 μM)	CYP1A
Phenobarbital (1000 μM)	CYP2B6

Rifampin (10 µM)	CYP3A

**Substrate (conc.)**	**Metabolite**	**Assay**
Ethoxyresorufin (2 µM)	Resorufin	CYP1A
Bupropion (200 µM)	Hydrozyl-bupropion	CYP2B6
Midazolam (10 µM)	1-hydroxymidazolam	CYP3A

Table S3: In vitro DMPK analysis: CYP induction. As indicated in Table S3, various compounds and substrates were applied as inducers of the investigated CYPs.

References for supplementary materials

1. Bowers, A.K.J.; Chow, E.; Xu, H.; Dror, R.O.; Eastwood, M.P.; Gregersen, B.A.; Klepeis, J.L.; Kolossvary, I.; Moraes, M.A.; ., F.D.S., et al. Scalable algorithms for molecular dynamics simulations on commodity clusters. In Proceedings of Proceedings of the 2006 ACM/IEEE conference on Supercomputing, Tampa, Florida; pp. 84–es.
